# User Profiles of a Smartphone Application to Support Drug Adherence — Experiences from the iNephro Project

**DOI:** 10.1371/journal.pone.0078547

**Published:** 2013-10-23

**Authors:** Stefan Becker, Andreas Kribben, Sven Meister, Clarissa Jonas Diamantidis, Nicole Unger, Anna Mitchell

**Affiliations:** 1 Department of Internal Medicine I, Marienhospital Herne, University Hospital, Ruhr University Bochum, Herne, Germany; 2 Department of Nephrology, University Duisburg-Essen, Essen, Germany; 3 Fraunhofer Institute for Software and Systems Engineering, Dortmund, Germany; 4 Division of Nephrology, University of Maryland School of Medicine, Baltimore, Maryland, United States of America; University of Groningen, University Medical Center Groningen, The Netherlands

## Abstract

**Purpose:**

One of the key problems in the drug therapy of patients with chronic conditions is drug adherence. In 2010 the initiative iNephro was launched (www.inephro.de). A software to support regular and correct drug intake was developed for a smartphone platform (iOS). The study investigated whether and how smartphone users deployed such an application.

**Methods:**

Together with cooperating partners the mobile application “Medikamentenplan” (“Medication Plan”) was developed. Users are able to keep and alter a list of their regular medication. A memory function supports regular intake. The application can be downloaded free of charge from the App Store™ by Apple™. After individual consent of users from December 2010 to April 2012 2042338 actions were recorded and analysed from the downloaded applications. Demographic data were collected from 2279 users with a questionnaire.

**Results:**

Overall the application was used by 11688 smartphone users. 29% (3406/11688) used it at least once a week for at least four weeks. 27% (3209/11688) used the application for at least 84 days. 68% (1554/2279) of users surveyed were male, the stated age of all users was between 6–87 years (mean 44). 74% of individuals (1697) declared to be suffering from cardiovascular disease, 13% (292) had a previous history of transplantation, 9% (205) were suffering from cancer, 7% (168) reported an impaired renal function and 7% (161) suffered from diabetes mellitus. 69% (1568) of users were on <6 different medications, 9% (201) on 6 – 10 and 1% (26) on more than 10.

**Conclusion:**

A new smartphone application, which supports drug adherence, was used regularly by chronically ill users with a wide range of diseases over a longer period of time. The majority of users so far were middle-aged and male.

## Introduction

Patients with chronic illnesses such as hypertension and chronic kidney disease are often burdened by high comorbidity and reduced awareness of their medical conditions, which creates a challenging environment in which to promote medication compliance [1]. Complexities of daily life, shifting priorities, and frequent poly-pharmacy likely contribute to patients' inability to deal adequately with their medical conditions [2]. Frequent encounters with the medical system, which result in dosage adjustments, add to the problems with medication compliance in these patients [3].

Novel tools are needed to address the requirements of these chronically ill patients. From a technical perspective, the “ideal system” would enable the user to easily 1) recognize and understand all necessary input components (i.e. drugs) and their changes, 2) treat these components as integral parts of their day to day activities and 3) record vital signs and possibly share them with the treating physician. The solution for such a tool is beyond the possibilities of a pencil, a piece of paper and an alarm clock.

“Mobile Computing”, which is a broad term comprised of various forms of hardware (i.e. smartphones, tablet computer) is presently one of the most important technological trends and offers promise for a “system solution” for patients with chronic disease [4]. It allows the user to download mobile applications (“apps”) via the Internet, which can then be applied for various activities of daily life [5]. In Germany the proportion of smartphones has increased rapidly over the past years and is now estimated to be a 55% market share of all sold cell phones [6]. Worldwide there were almost 6 billion mobile phones being used in late 2011, more than one billion of these had broadband capabilities and 43.6 billion mobile applications were downloaded in the 12 months ending September 2012 [7], [8]. With the global prevalence of mobile technology, accessing health-related applications via mobile phone seems a logical step in the patients' management of their own medical conditions [7]. According to a US study, 25% of smartphone users were already using such health applications and almost half of those asked would be interested in doing so [9]. In this paper, we introduce a novel mobile application called “Medikamentenplan” (“Medication Plan”), which was developed to support medication compliance and vital sign documentation. We describe its implementation and acceptance in a representative German population subset. Pre-specified main endpoints of the statistical analysis were the frequency and intensity of use and the users' demographic characteristics.

## Materials and Methods

### Ethics

The Ethics Committee of the Medical Faculty of Essen University was consulted and a formal written waiver for the need of ethics approval was issued (13-5373-BO).

**Figure 1:**
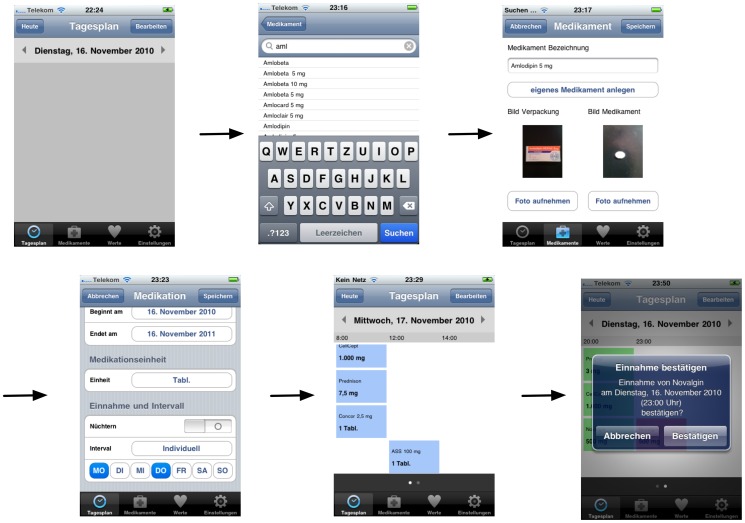
Generating a medication plan on the smartphone (exemplary screenshots).

In 2010 the “Medication Plan” application was specified for iOS by the Department of Nephrology, Essen University Hospital, Essen, Germany, in cooperation with the companies DigitalOffice (Dortmund, Germany), Bergisch Media (Heiligenhaus, Germany) and Digitalpocket (Bochum, Germany) and the support of the German Society of Nephrology. “Medication Plan” is a native smartphone application, which allows users to maintain and alter a drug therapy plan on their personal device (fig. 1). An online drug database customized by the Department of Nephrology at Essen University Hospital, facilitates the entries. Users may specify intake requirements according to the medication regimen issued by the prescribing physician and the patients' own personal needs (e.g. how long to take the medication, medication dosage, whether to take on an empty stomach, how often to take, and at what time). A memory function and local push-notification alert remind users to take their medications at the pre-specified time. No permanent Internet connection is necessary and all data is stored locally on the device itself, reducing the possibility of erroneous transmission of personal health information. Users can enter vital sign data, and trends are presented graphically. If required, data can then be attached as csv-files and e-mailed to third parties.

The application was released first as a 1.0 version in the German-language App Store™ by Apple™ on December 14, 2010 and was available for download free of charge (http://itunes.apple.com/de/app/inephro-medikamentenplan/id405270576?mt=8 #). The application was actively advertised by the German Society of Nephrology (DGfN) by press releases and on its website (http://www.dgfn.eu/aktuell/inephro.html). This triggered a series of further articles in newspapers, television, patient self-help groups and blogs [10]–[15]. Interestingly after a positive article by a renowned consumer protection association (“Stiftung Warentest”) a significant increase of downloads could be detected [11]. Three additional updates were issued, which included enhancement of the medication specification requested by a large number of users, as well as the removal of software “bugs”. Prior to using the application a disclaimer as well as an agreement for a subsequent anonymous analysis of user data had to be consented by the user via activation of a hyperlink (implementation by QUEST objects GmbH, Tübingen). Additionally users were invited to voluntarily and anonymously fill in an online questionnaire, which had to be actively accessed by another hyperlink. The unique identifier numbers (UDID) of the respective iPhones were irreversibally encrypted by a MD5 message-digest algorithm (MD5-Hash). The activitiy of the encrypted UDID was then tracked (“creation”, “modification”, or “deletion” of drug information, as well as the “confirmation” of drug adherence within the application “Medication Plan”). Activity of the respective, encrypted UDID addresses and if available, associated demographic information were analysed using self-implemented software of Fraunhofer ISST, Dortmund, Germany, and GraphPad Prism version 6.0 for Macintosh, GraphPad Software, San Diego California U.S.A. Chi-square Test was performed by VassarStats (http://vassarstats.net/odds2×2.html). Data are presented as descriptive statistics and relative values were calculated using the corresponding sample.

## Results

**Figure 2:**
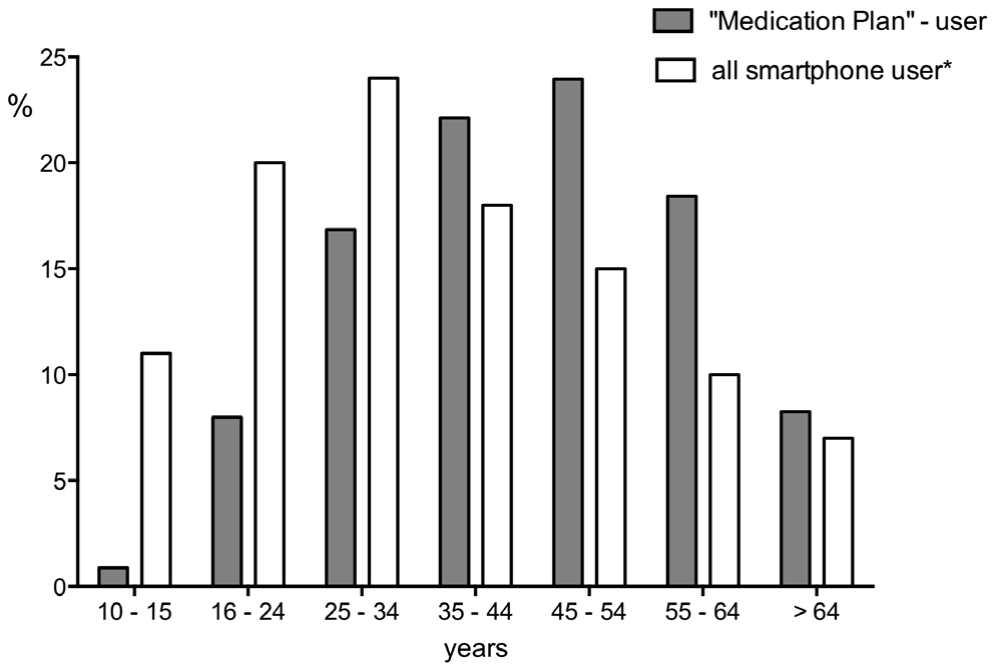
Relative proportion by age of users of "Medication Plan" who provided demographic data (n = 2279) and all smartphone users (*Federal Statistical Office, 2011 [11]).

**Figure 3:**
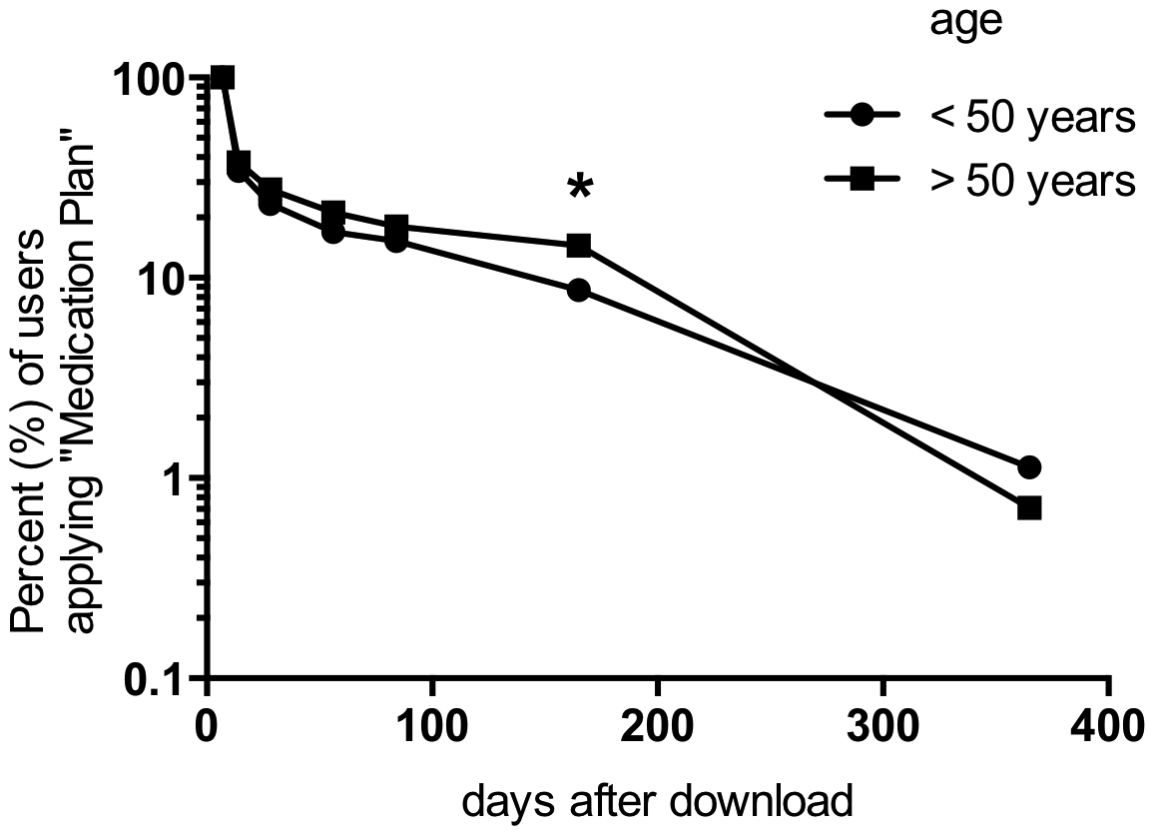
1095 regular users (defined as applying "Medication Plan" at least once a week for at least 28 days) specified their age. At 28 days after download 23% (124/530) of users <50 years and 28% (156/565) of users >50 years were still using the application at least once a week (p>0.05). The proportion of older individuals using “Medication Plan” at 165 days was significantly higher (<50 years: 9% (46/530) and >50 years 15% (82/565); p<0.004*). After 365 days only 1% of regular users <50 years (6/530) as well as >50 years (4/565) were using the application.

As of April 2, 2012, “Medication Plan” was downloaded and used by 11688 smartphone users. 29% (3406) used the application at least once a week for at least 28 days and in 27% (3209) of cases any activity was recorded for at least 84 days (table 1). 19% (2279) of users provided demographic data via the anonymous online questionnaire. 68% (1554) of survey respondents were male. 49% (1122) of users had finished secondary school as highest educational qualification, 18% (420) had finished school with qualifications for university studies and 26% (589) were holding a university degree (table 1). The majority of users were suffering from cardiovascular disease (table 2). The stated age of all users was between 6–87 years (mean 44). 27% of users were 55 years or older (data from 16, fig. 2). 1095 Regular users (defined as applying the software at least once a week for at least 28 days) deployed the software temporarily, and specified their age (fig. 3). At 28 days after download 23% (124/530) of users <50 years and 28% (156/565) of users >50 years were still using the application at least once a week (p>0.05). The proportion of older users applying regularly “Medication Plan” at 165 days was significantly higher (<50 years: 9% (46/530) and >50 years 15% (82/565); p<0.004). After 365 days only 1% (6/530) of users <50 years and >50 years (4/565) were regularly using the application (fig. 3). Out of 2279 users approximately two thirds (69%; 1568) were taking 1 – 5 different medications a day. 9% (201) stated to be taking 6 – 10 and 1% (26) were on more than 10 drugs per day. Interestingly, 21% (484/2279) were not on any medication at all. From 196 feedback e-mails 68% (134) presented ideas for further development, 28% (55) referred to software bugs and 4% (7) were inquiries on software function. Table 3 summarizes the different suggestions and requests for further development.

**Table 1:** User activity over time and demographic characteristics in smartphone users, who downloaded and used "Medication Plan from 12/14/2010 - 04/02/2012 (* regular use was defined as using the application at least once a week for at least 28 days, § percent of 2279 users who provided personal information).

n (%)	User activity and demographic characteristics
11688	Total users
3406 (29)	Regular users*
3209 (27)	Application used for at least 84 days
2279	Users who provided personal information
1554 (68^§^)	Men
1122 (49^§^)	Finished secondary school as highest educational qualification
420 (18^§^)	Finished school with qualifications for university studies as highest educational qualification
589 (26^§^)	Holding a university degree as highest educational qualification

**Table 2:** Diseases declared by users (multiple entries possible).

Disease	n = 2279 (%)
Cardiovascular diseases	1697 (74)
History of transplantation (e.g. kidney or liver)	292 (13)
History of cancer	205 (9)
Impaired renal function	168 (7)
Diabetes mellitus	161 (7)
Lung disease	105 (5)
Liver disease	105 (5)
Diseases of the gastrointestinal tract	61 (3)

**Table 3:** Topics of Feedback-Mails proposing further development of the application.

Topic of e-mails	n = 134
Proposals for further specification of drug plan	60
Would the app also be available on Android?	14
Would it be possible to document relevant laboratory values and physical signs such as pain and temperature?	11
Would it be possible to export vital sign parameters in different formats (i.e. PDF)?	9
Would it be possible to implement a diary function (i.e. heart rhythm disturbances)?	6
Would it be possible to comment on drugs?	5
Would it be possible to keep stock of medication?	4
Would it be possible to better choose the of push – notification bleep?	4
Would it be possible to implement a zoom function?	3
Is the app available for other smartphones?	3
Would it be possible to import the drug information via a barcode?	3
Would it be possible to entre data on weight more exactly?	2
Are different user profiles possible?	2
Would it be possible to keep a history of medications?	2
Would it be possible to make the app available for Windows/Blackberry – Smartphones?	2/1
Would it be possible to synchronize the app with other devices (i.e. smartphone/tablet)?	1
Would it be possible to create a backup of drug data?	1
Would it be possible to connect the application with the hospital information system?	1

## Discussion

The present study is the first to describe the implementation of a mobile application to support drug adherence published in a public app–store. We analysed frequency and intensity of the app's use and also evaluated the demographic profile of its users.

### Brief review of findings and comparison to prior studies

The overall interest in the app of more than 11000 users was remarkable. However, download rates of other health-related apps, which targeted individuals with acute rather than chronic medical conditions have been reported to be much higher [7]. A software targeting chronically ill patients may at present only draw limited public interest, likely due to the epidemiological factors discussed below. Also, the application was published only in the German-language App Store™ by Apple™, which undoubtedly restricted its reach. Today there is still a threefold “digital divide” between age groups, sexes and according to education levels: Currently, the majority of German smartphone users are younger than 35 years although use in individuals older than 45 years is increasing steadily [16]. This is similarly reflected in the US trend of usage [17]. Compared with the average age of German smartphone users, the users of “Medication Plan” were older [16]. Two factors, likely, have contributed to this. Firstly it seems natural, since the prevalence of chronic diseases like hypertension or chronic ischemic heart disease grows with age [18]: In Germany 49% of women and 41% of men between 45 and 65 years declare to be suffering from chronic conditions [19]. More than half of the population are affected in the age cohort above 65 years [19]. Secondly, the German Society of Nephrology and several self-help groups actively advertised or reported on the application. This will probably have helped to draw the attention of older users. The fact that more than two thirds of our users were male illustrates the “digital divide” between the sexes. Women in general are still less interested in using the mobile Internet than men, as shown by surveys both from the German federal statistics institute and data from the US [5], [16]. In Germany at present especially older women use the mobile Internet significantly less than men [16]. A further schism exists between individuals with higher education and those with only basic qualifications. Among our users the proportion of university graduates was higher compared to the general population (28% vs. 13%) [20].

Regular use of the application decreased considerably within the first 2 months and lasted for more than a year only in a few cases. This finding is consistent with other studies citing a high attrition rate for internet interventions [21], which may be a reflection of an early interest in the novelty of the application, with a decline in eagerness as the newness of the intervention wears off. At present there is no scientific data on why people stop using mobile health applications. In a commercial survey by the Consumer Health Information Corporation (CHIC) on the use of healthcare apps in 2011 users reported "not user friendly" and "found a better one" as the most frequent reasons for no longer using an app (32% and 34% respectively). 26% of health apps were used only once after download. The drop out rate recorded in this survey was 74% (of 395 participants) by 10th use [22]. The fact that "Medication Plan" was not used permanently might therefore reflect deficits in usability and the necessity to continuously monitor customer/patients' demands. A criticism in usability might be that the app requires to set end dates for the medication. However, the aspect of an “unlimited therapy duration” was not raised in feedback mails. Others have found that most patients value interactive systems, i.e. devices that will give feedback - in this case on correct drug intake, a feature as yet not included in "Medication Plan" [23], [24]. Also, patients seem to prefer to use m-health in conjunction with visits to their doctors - another element which was not part of our study [23], [24]. In essence, use of this app can be seen as an expression of patients' wishes to improve the effectiveness of their treatment by improving their adherence. The most favourable - if speculative - explanation for the decline in use of "Medication Plan" is that it may only have been used temporarily as a "learning tool" at least by some patients: Once habituation to correct drug intake had been achieved, assistance may no longer have been regarded as necessary. Following this train of thought the fact that older individuals seemed to be using the app for a longer period than younger users may reflect a lesser slope in the learning curve of older users and the temporary adoption of the app as a "companion tool". Furthermore experiences from an “Ambient Assisted Living Project”, which is dedicated to the needs of an older group of users, report that the acceptance of 7-inch tablet PCs is very high [25]. In this context further studies should explore the possibilities of tablet computers for elderly and multimorbid users and patients' motivation for using an app like "Medication Plan". In summary, most of the users seem to have been “early adopters” of a new service: middle-aged male and - derived from the relatively small number of daily taken pills - comparatively healthy [26].

## Conclusion

Use of the Internet and of smartphones to deliver health care is growing rapidly. Electronic devices are increasingly used by both health care providers and patients as communication tools [27]. Providers using mobile communication technology to connect with their patients achieve an improved overall patient-provider communication, strengthened patient autonomy, and empowered patients to tackle daily health issues [28]–[33]. Studies from various clinical contexts, in which text-messaging services were introduced, reported improved medication adherence [34]–[39]. They also showed that electronic self-documentation can be an effective tool in the management of chronic conditions (i.e. self measurement of blood glucose in diabetes patients) [40].

In the present climate it seems likely that health-related mobile computing applications will increasingly play a role for elderly and chronically ill users for a variety of reasons. Currently, most of the hardware and commercially successful applications are likely to be targeting younger (between 18 – 40 years) and healthy individuals because they constitute the majority of contemporary smartphone users [5], [16]. However, the young and middle-aged of today are the senior citizens of tomorrow. This cohort effect will alter the demographics of those who rely on information technology and who increasingly integrate the mobile internet into their daily lives [7], [41].

Future developments should take into consideration that generally Internet use is inversely correlated with an increasing number of medical conditions and lack of financial resources [42]. Also, a decrease of associated costs of mobile devices, i.e. costs for smartphones and tablet computers, may be essential to mitigate the “digital divide": Others have suggested that a portion of the population on the socioeconomic margin may miss out on the benefits of technologies due to prohibitive factors such as device cost [43], [44]. This is supported by the suggestion of "Medication Plan"-users to publish a similar application for other, possibly cheaper systems, as borne out in the mail requests.

In many cases the choice of consumers to download our application seems to have been promoted by media recommendations. This reflects the element of novelty in apps like "Medication Plan". We believe that some form of guidance and possibly coaching by the medical profession will - for the time being - be necessary to establish health-related apps as a fixture in patient care. In order to ensure that relevant technical standards are met, one may discuss whether health care-related apps generally require a conformity assessment and how to best communicate quality standards to consumers.

Many ideas of our users arose around the problem of importing their individual medication plan into the smartphone. Our vision for the future involves an automatic transfer of therapy plans issued by physicians onto mobile devices via electronic health records. This would promote acceptance and decrease the number of associated transmission errors. Applications will have to be tailored closely to the specific demands of sick individuals to be accepted as part of their often complicated day-to day routines [45], [46]. Not overloading an application with functionality seems vital, when developing for elderly users with chronic conditions. A low-threshold service to allow a provider-patient connection with a smartphone application could be a promising approach.

Our data allow a fleeting glimpse into the future, where "mobile health" will not replace the doctor-patient relationship, but will hopefully help to establish more effective and efficient treatment and accelerate e-health strategies.
